# Introducing the NUATEI Consortium: A Mexican Research Program for the Identification of Natural and Synthetic Antimicrobial Compounds for Prevalent Infectious Diseases

**DOI:** 10.3390/ph17070957

**Published:** 2024-07-18

**Authors:** Julio César Carrero, Bertha Espinoza, Leonor Huerta, Mayra Silva-Miranda, Silvia-Laura Guzmán-Gutierrez, Alejandro Dorazco-González, Ricardo Reyes-Chilpa, Clara Espitia, Sergio Sánchez

**Affiliations:** 1 Departamento de Inmunología, Instituto de Investigaciones Biomédicas, Universidad Nacional Autónoma de México, Mexico City 04510, Mexico; besgu@iibiomedicas.unam.mx (B.E.); leonorhh@biomedicas.unam.mx (L.H.); espitia@biomedicas.unam.mx (C.E.); 2 CONAHCyT-Instituto de Investigaciones Biomédicas, Universidad Nacional Autónoma de México, Mexico City 04510, Mexico; msilvami@conacyt.mx (M.S.-M.); saguzmangu@conacyt.mx (S.-L.G.-G.); 3 Departmento de Química Inorgánica, Instituto de Química, Universidad Nacional Autónoma de México, Mexico City 04510, Mexico; adg@unam.mx; 4 Departamento de Productos Naturales, Instituto de Química, Universidad Nacional Autónoma de México, Mexico City 04510, Mexico; chilpa@unam.mx; 5 Departamento de Biología Molecular y Biotecnología, Instituto de Investigaciones Biomédicas, Universidad Nacional Autónoma de México, Mexico City 04510, Mexico; sersan@biomedicas.unam.mx

**Keywords:** consortium, NUATEI, natural products, anti-infective agents, tuberculosis, amoebiasis, trypanosomiasis, HIV

## Abstract

The need for new drugs to treat human infections is a global health concern. Diseases like tuberculosis, trypanosomiasis, amoebiasis, and AIDS remain significant problems, especially in developing countries like Mexico. Despite existing treatments, issues such as resistance and adverse effects drive the search for new alternatives. Herein, we introduce the NUATEI research consortium, made up of experts from the Institute of Biomedical Research at UNAM, who identify and obtain natural and synthetic compounds and test their effects against human pathogens using in vitro and in vivo models. The consortium has evaluated hundreds of natural extracts and compounds against the pathogens causing tuberculosis, trypanosomiasis, amoebiasis, and AIDS, rendering promising results, including a patent with potential for preclinical studies. This paper presents the rationale behind the formation of this consortium, as well as its objectives and strategies, emphasizing the importance of natural and synthetic products as sources of antimicrobial compounds and the relevance of the diseases studied. Finally, we briefly describe the methods of the evaluation of the compounds in each biological model and the main achievements. The potential of the consortium to screen numerous compounds and identify new therapeutic agents is highlighted, demonstrating its significant contribution to addressing these infectious diseases.

## 1. Introduction

Infectious diseases such as tuberculosis, trypanosomiasis, amoebiasis, and AIDS are some of the most important pathologies affecting the world, particularly in developing countries such as México [1] (Table 1). The control and eradication of these diseases are subject to numerous social factors involving economic, educational, and public health aspects, as well as factors inherent to the host and the infectious agents. Added to this is the recent emergence of pandemic microorganisms such as the SARS-CoV-2 virus, the causative agent of the COVID-19 pandemic, which has posed a complication for the diagnosis and treatment of other infectious diseases, temporarily relegated to the background [2].

The current development of new drugs creeps far behind the outburst of infectious diseases and cancers but, above all, the development of multidrug resistance. Therefore, finding novel products to face this problem is urgent. Natural products have a substantial beneficial potential for humanity as they are the source of most drugs in clinical use today, mainly secondary metabolites, which is especially true in the case of anti-infective agents [9]. Accordingly, 50% to 65% of our pharmaceutical drugs are directly or indirectly related to natural products, including biologically or chemically modified natural products (semisynthetic derivatives) [10].

Due to the characteristics of virulence, pathogenicity, and genetic variability of infectious pathogens, and very importantly, the drug resistance they are permanently developing [11], there is a need for a continuous and systematic search for new therapeutic agents. In this regard, natural products continue to be a cost-effective tool in drug development. Part of the success of studies on natural products lies in the fact that they have taken advantage of the technological development of tools that reduce the isolation of compounds with redundant structures, including the use of computational platforms to predict new biological activities of already-known molecules [12]. Furthermore, knowledge about pathogenic microorganisms and their infection mechanisms has been increasing, so new targets of action are known, which opens a window of possibilities for already known and new natural products to expand their scope of action. Another option for the use of natural products is to combine them with the drugs of choice for the treatment of infections, allowing the required dose of the antibiotic to be reduced, minimizing its side effects, and reducing the probability of developing resistance [13]. Moreover, natural plant products can act on virulence factors of pathogenic microorganisms, allowing antibiotics to exert their effect more efficiently [14]. On the other hand, there are chemically synthesized compounds in laboratories, which are usually strongly inspired by mimicking the effects of natural drugs [15]. The first development of specific artificial drugs to combat infectious diseases began to assume its modern form about a century ago [16]. In principle, these synthetic drugs are designed to combat the most lethal parasitic, bacterial, or viral infections, which involve several mechanisms of action.

In light of the global importance of the above-mentioned infectious diseases and the interest of our research groups, we have embarked on establishing an institutional consortium called NUATEI (New Alternatives for the Treatment of Infectious Diseases, for its acronym in Spanish), formed by a group of multidisciplinary experts in different areas of knowledge, such as microbiology, parasitology, virology, and immunology, together with chemists specialized in obtaining drugs derived from plants and synthetic compounds. The consortium was formed 7 years ago within the framework of the Institutional Research Programs of the Institute of Biomedical Research of Universidad Nacional Autónoma de México (UNAM), which encourages collaborative work and allocates resources to address innovative projects with an impact on public health, developing strategic projects by bringing together intellectual and infrastructure capacities. The NUATEI consortium’s main objective is the development, evaluation, and identification of compounds of natural and synthetic origin that affect the viability and infectivity of the pathogens responsible for four of the most prevalent infectious diseases of México and developing world: tuberculosis, Chagas disease, amoebiasis, and HIV (Table 1).

In this paper, we describe the details of how the consortium operates, the generalities of compounds of natural and synthetic origin, details regarding each of the biological models evaluated and the strategies that we have followed to identify promising compounds, and a summary of the most relevant results obtained by the consortium so far, most of them previously published but shown here together to dimension the scope of the NUATEI program.

## 2. Consortium Operating Chain

The consortium works following a simple scheme, which begins with the acquisition of a compound and ends, in the first phase that we report here, with tests in the viability/infectivity of the infectious agents of the four biological models studied: tuberculosis, Chagas disease, amoebiasis, and AIDS. The studied molecules can have multiple origins, such as natural or synthetic, and are distributed among the working groups to be evaluated for their ability to affect the viability or infectivity of each of the microorganisms. The consortium has worked mostly with plant extracts and their secondary metabolites and, to a lesser extent, with endophytic compounds from fungi and bacteria and antimicrobial molecules of mammalian origin. The incentive to work with plants comes from the fact that the biodiversity of the Mexican flora is very broad, with an estimated 23,424 native vascular species [17]. Approximately 3300 of these are currently used medicinally by 54 ethnic groups, peasants, and even urban populations [18]. In addition, Mexican traditional medicine can be tracked historically in books written along centuries, such as *The Codex Cruz-Badiano* of 1552, which compiles native medicinal plants used in the XVI century and presumably in pre-Columbian times [19]. Therefore, Mexican flora can be recognized as an important source of natural products with the potential to develop drugs [20,21,22]. The history of drug development has shown that natural products are a promising option, either in their original form or taken as models for the semi-synthesis or synthesis of new compounds [9]. On the other hand, we have also studied the antimicrobial effect of some compounds obtained by chemical synthesis with a structural design specifically directed at certain pathogen targets and compounds from databases identified through computational chemistry.

Most extracts and compounds used are prepared in laboratories of the UNAM Chemistry Institute (Dr. Ricardo Reyes-Chilpa and Dr. Roberto Martínez), which collect plants recognized by traditional Mexican medicine as specimens with antimicrobial properties. The selection of the study plants is carried out using ethnomedical and chemotaxonomic criteria. Once the phytoextracts with activity have been identified, the active ingredients responsible for the antimicrobial effect are determined and purified. Only the active extracts are separated into a few fractions whose biological activity is re-evaluated, and only the most active fractions are chemically studied. Usually, GC-MS is carried out for the putative detection of the compounds in the slightly polar active extracts. If necessary, new compounds are designed based on the active ingredients, or existing ones are modified through chemical synthesis. Metabolites derived from plant-associated microorganisms (endophytes) are purified from cultures and are contributed to the consortium by one of the IIB-UNAM groups (Dr. Sergio Sánchez) and by the group of Dr. Iván Ortega Blake from the Institute of Physical Sciences-UNAM. A group from the Autonomous University of Sinaloa (Dr. Nidia León-Sicairos) has collaborated by providing bovine lactoferrin and synthetic peptides derived from it, an antimicrobial protein from breast milk and neutrophil granules that have powerful antimicrobial properties [23]. The groups of Drs. Daniel Chávez Velasco from the Tijuana Institute of Technology, José Luis Medina-Franco from the Faculty of Chemistry, and Alejandro Dorazco from the Institute of Chemistry (all of them at UNAM) have contributed to the synthesis of new molecules through computational chemistry or by modifying compounds existing on the market.

**Figure 1:**
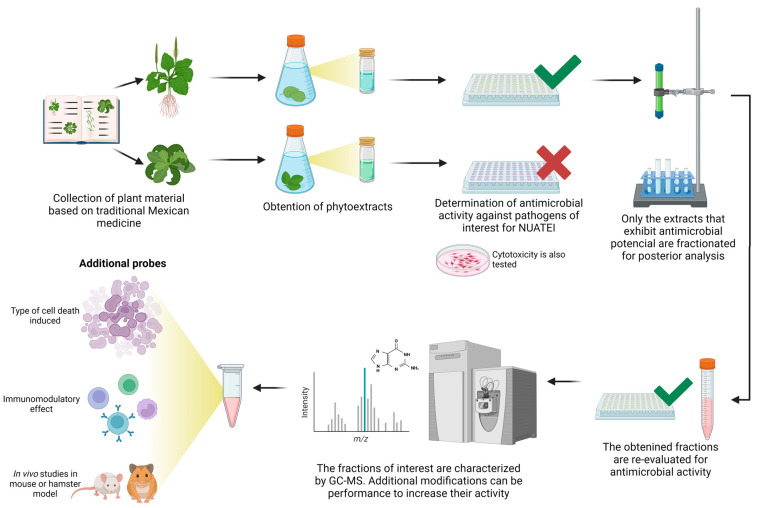
General scheme for the evaluation of plant-derived products against infectious microorganisms in the NUATEI consortium. Image was made with BioRender.com.

Once an extract or purified compound is selected, its in vitro cytotoxic activity against mammalian cells is first evaluated. These assays are carried out in cell lines such as HepG2 or, in our case, VERO cells following criteria that have been previously proposed for the identification of new drugs against infectious agents in the developing world [24]. Then, the samples are distributed to the different research groups to evaluate their in vitro activity against pathogens, particularly their effect on viability, replication, and, in some cases, the type of death induced (see details for each biological model below). When promising results are obtained, the compound is tested on in vivo models of the diseases, mostly rodents such as mice or hamsters (Figure 1). For several of the compounds evaluated against amoeba and Trypanosoma, studies have been carried out on the mechanisms of action, including the type of stress, the type of cell death induced (apoptosis or necrosis), or immunomodulation studies (Figure 1). Finally, in the case of those compounds having outstanding characteristics, such as good antimicrobial activity, high biocompatibility, and good pharmacological disposition, the possibility of protecting the compound by patenting is considered, a process that involves specialized organizations in the matter, such as the UNAM Liaison Office and the Mexican Institute of Industrial Protection (IMPI). Furthermore, the identification of new compounds with low cytotoxicity and high activity against infectious organisms in preclinical studies will encourage the application of the consortium for financial support for future clinical trials.

## 3. Natural Products: Overview

Natural products are substances biosynthesized by living organisms, including those arising from primary and secondary metabolism. The treatment of infectious diseases has focused on secondary metabolites such as alkaloids, phenylpropanoids, terpenes and their derivatives [25,26]. From 1981 to 2019, the FDA approved 1881 drugs, of which 1394 were small molecules. Sixty-six percent of these drugs were natural products, semisynthetic or synthetic compounds that are inspired by or imitate a substance produced by plants, microorganisms, or even animals [9]. Currently, research into natural products has been favored by the computational methods that allow the identification of the most promising natural products for extraction, purification, (partial) synthesis, and biological testing for drug discovery, including ADME, toxicity, and target prediction [27].

### 3.1. Antimicrobial Compounds from Plants

Natural products from plants have been successful as a source of antiparasitic compounds. The most notable examples are the antimalarial drugs artemisinin from *Artemisia annua* (Asteraceae) and quinine from *Cinchona succirubra* (Rubiaceae) [28]. Since malaria is considered the deadliest parasitic infection in the world [29], the importance of such a discovery is evident, as it has saved millions of lives since its introduction, especially on the African continent. In fact, the discovery of artemisinin earned Dr. Tu Youyou the Nobel Prize in Physiology or Medicine in 2015 [30]. Regarding antiviral drugs, the most recent success stories have been compounds prepared by chemical synthesis and inspired by natural products, such as the drug grazoprevir, an inhibitor of the NS3/4A protease of genotype 1 of the hepatitis C virus (HCV) [9].

Taking advantage of the natural resources of our country, the two criteria used by our research consortium to select sources with potential antimicrobial activity are ethnopharmacological and chemotaxonomic. Regarding the ethnomedical criterion, the valuable information that traditional medicine provides around the world is currently recognized. Thus, the identification of many active phytocompounds, such as the case of artemisinin mentioned above, was achieved thanks to information recorded almost 1800 years ago in texts of traditional Chinese medicine [31]. This encouraged the WHO to promote the study of different elements used in traditional medicine, including medicinal plants, through the founding of the “WHO World Center for Traditional Medicine” [32]. In this regard, for the NUATEI consortium, we have reviewed promising plant species in databases such as “Enciclovida”, “CONABIO”, “Repositorio del Patrimonio Cultural de México”, “Digital Library of Traditional Mexican Medicine”, and “National Digital Library of Mexico and the Tropics”, among others. On the other hand, the chemotaxonomic strategy is related to the selection of species belonging to the taxa (genus, tribe) from which active metabolites have previously been obtained. For example, our consortium has studied the antiviral and antifimic activity of the species *Calophyllum brasiliense*, which belongs to the same family as *Calophyllum lanigerum*, a source of antiviral compounds [33,34].

### 3.2. Antimicrobial Compounds of Microbial Origin

Microorganisms such as bacteria and fungi are potential factories of many bioactive natural products, some highly valuable to humankind [35]. Many secondary metabolites have been successfully isolated and identified from microorganisms with potent antimicrobial properties, such as the antibiotics tetracycline, streptomycin, and erythromycin, all isolated from filamentous fungi of the genus *Streptomyces* [36]. In 2015, almost 29,000 antibiotics were reported to be of microbial origin [37], and only in 2019 did the FDA approve the use of cefiderocol, an antibiotic with a cephalosporin-based structure discovered 70 years ago [38]. Of all microbial antibiotics, approximately 40% are of fungal origin, 40% come from actinomycetes, and 20% come from unicellular bacteria [39]. The distribution of microbial molecules varies in different ratios from the microorganism isolated. Cyanobacteria and eubacteria produce more bioactive peptide-type molecules (62% and 53%, respectively) than others. Fungi generate more terpenoids (18%), and some actinobacteria make a lot of macrocyclic lactone compounds (27%). A few drugs are produced only by specific microorganisms. Thus, some *Streptomyces* species produce tetracycline-type antibiotics, echinomycin-type compounds, quinoxaline peptides, and polyene macrolides. Some fungi can synthesize brefeldin, cytochalasins, condensed macrolides, gliotoxin-like diketopiperazines, peptaibol peptides, and heterocyclic compounds as simple pyrones [37]. Unfortunately, only 1.5% of the actual actinobacteria and about 5% of the known fungi are cultivable by current methodologies, limiting the possibility of isolating new species. However, microbes are always notable, as one species may contain 10 to 20 unexpressed biosynthetic clusters. Breaking points were papers describing the complete genome sequence from two Gram-positive bacteria, *Streptomyces coelicolor* and *Streptomyces avermitilis*, which warned the scientific community that secondary metabolite production by these bacteria had been long underestimated [40,41]. Until now, over 130,000 complete genome sequences from more than 50 phyla are available in one or more scaffolds [42], and only over a hundred belong to fungi. Third-generation sequencing platforms and sequence analysis using online and stand-alone software tools are helping to predict the presence of genes or gene clusters encoding for antibiotics and other secondary metabolite production.

Notably, the main new places to start searching for novel antimicrobial-producing microorganisms are oceans and endophytic organisms from plants or similar living things. There is a relatively new trend of investigating some 40 million insect species and their products [43,44], which may result in the discovery of new chemicals. However, the rediscovery of previously studied strains and known compounds has significantly increased with the accumulation of strains and compounds over the years. The above examples prove that natural products continue to be a profitable tool in drug development.

## 4. Antimicrobial Synthetic Compounds

The design of synthetic compounds has several obvious advantages over the search for natural compounds, including the following: (1) they have a customized molecular design against the target parasite/bacteria/virus; (2) they do not require the collection of biological material; (3) their manufacturing is timeless and has geographical flexibility; (4) they can be produced on a large scale; (5) they can decrease or eliminate biological contamination; and (6) the can be designed for generating compounds under the premise of high stability for storage and distribution, among others. Despite the foregoing advantages, these synthetic drugs still present critical drawbacks associated with their low efficacy and severe toxic side effects, such as fever, rash, hypersensitivity, generalized edema, joint/muscle pain, bone marrow suppression, and lymphadenopathy [45]. In principle, many of these disadvantages can be overcome through the chemical modifications of commercial drugs, such as the insertion of hydrophilic groups to increase their solubility and stability in physiological media, which should potentially increase their activity [46].

The approach strategies for the design of new compounds are very varied. For example, to mention a few, more powerful, less toxic, and more soluble analogs can be developed from the structure of compounds with already proven activity or those that are in use as treatment in humans. Various software tools can also be used for the in silico identification of synthetic compounds present in databases that bind with high affinity to the target molecules recognized in pathogens. Similarly, computer programs can be used for the design of small molecules with presumed antimicrobial activity. For example, at present, the only pharmaceutical drug worldwide options for Chagas disease are a synthetic nitroimidazole ring-based drug, benznidazole (Bz), which has been in use now for over 50 years, and a nitrofuran-based drug, nifurtimox (Nfx), which only works in the acute phase of the disease [47]. An à la carte molecular design of these low-weight compounds includes a nitro group in the heterocyclic rings. Studies have shown that the activities of Bz and Nfx are due to the release of nitro-anion radicals that damage the parasite DNA [48]. Discovery of this structure–activity relationship was achieved through rational design and chemical synthesis. The general picture of the discovery of synthetic drugs against *Entamoeba histolytica* and *Mycobacterium tuberculosis* is similar. The amoebiasis and tuberculosis drugs currently in use were developed over 60 and 40 years ago, respectively. However, in spite of their efficacy, a lengthy treatment duration and side effects increase their toxicity for humans.

The possibilities of drug design are very wide; however, we will focus here on the small synthetic drugs, as several groups in the NUATEI consortium are using this approach. Small synthetic molecules are typically cheap to manufacture and have better storage, stability, and physiological distribution properties than large molecules. Identifying small new synthetic compounds active against specific molecular targets can be supported by developing large information libraries containing the physicochemical features of protein–ligand interactions. The last decade has seen the enormous and still growing field of chemoinformatic tools and the development of computational techniques for the detailed analysis of the relationship between chemical structure and biological activity (structure–activity relationship, or SAR) for drug discovery. A SAR study implicates the elaboration of a series of chemical modifications of a core structure associated with a defined increase or decrease in biological activity so that if there are multiple structures, a model of SAR may guide the search for new active candidates [49]. Originating in the 1960s, quantitative structure–activity relationship (QSAR) modeling has become an integral tool in medicinal chemistry. Modeling using QSAR has the purpose of deriving linear mathematical models that relate combinations of molecular numerical descriptors to the biological activity of a series of compounds (potency values), with the ultimate objective of predicting new and more potent analogs. Thus, QSAR is focused on identifying substitutions in analogs that are most critical for their bioactivity in the context of the whole chemical interactions with the active site of a macromolecule. Understanding protein–ligand interactions leading to drug discovery requires the efficient analysis of the structural information of protein–ligand complexes (crystal structures) and the combination of molecular modeling (detailed description of atomic interactions) with chemoinformatic approaches (analysis of databases of experimentally tested compounds for the construction of structure–function landscapes). Molecular modeling techniques transform atomic interaction information into three-dimensional representations and include molecular mechanics, quantum mechanics, molecular dynamics and pharmacophore modeling. Instead, chemoinformatic methods transform the atomic interaction information into two-dimensional (2D) or one-dimensional (ID) representations of that information to allow its rapid and easy visualization, clustering, and mining [50].

Such studies, initially descriptive, can be used in a predictive manner and as tools for the design of new synthetic compounds or the identification of potential new applications for already existing ones. Ultimately, the proposed compounds must still be synthesized and then udergo real experimental testing to decide their potential effectiveness. The results of the experimental tests and the computational analysis feed into each other to improve the efficacy of the proposed synthetic compounds [51].

## 5. Biological Models of Infectious Diseases Targeted by the NUATEI Consortium

**Figure 2:**
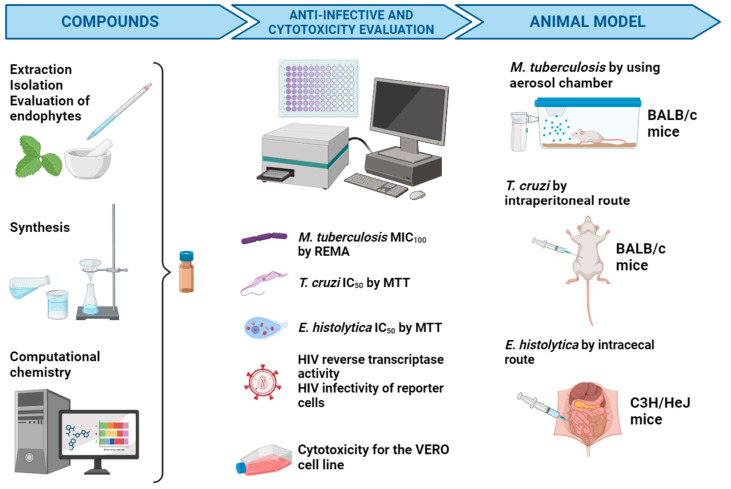
Workflow of the NUATEI consortium for the identification of new antimicrobial compounds. Imagen was made with BioRender.com.

Once the compounds to be evaluated are selected (whether they are purified from plants, endophytes, or animal sources, chemically synthesized, or selected by computational chemistry), they are evaluated in the first instance to determine their cytotoxic potential against mammalian cells and their in vitro antimicrobial potential against four infectious agents: *M. tuberculosis*, *T. cruzi*, *E. histolytica*, and HIV. Only those compounds showing a combination of high antimicrobial activity with low cytotoxicity move to the second stage, where their potential to inhibit the establishment of infections in animal models is evaluated (Figure 2).

Next, each of the four biological models evaluated by the NUATEI consortium will be introduced, including a brief description of the methodology used for the evaluation of the compounds and the most relevant results in each case.

### 5.1. Tuberculosis as a Target of the NUATEI Program

According to WHO, tuberculosis (TB) remains a major disease in terms of mortality and morbidity, with a global estimation of close to 1.3 million deaths and 10.6 million cases of disease in 2022 [52]. Among tuberculosis cases, 6.3% were among people living with HIV. Geographically, most TB cases in 2022 occurred in the WHO regions of South-East Asia (46%), Africa (23%) and the Western Pacific (18%), with smaller shares in the Eastern Mediterranean (8.1%), the Americas (2.9%), and Europe (2.2%) [53]. The etiological agent of tuberculosis, *Mycobacterium tuberculosis*, is an acid-fast bacillus (AFB) that infects humans through the inhalation of droplets containing bacteria and develops mainly a pulmonary infection [54].

Close contacts of tuberculosis cases are susceptible to becoming infected and progressing to active disease, particularly during the first year after exposure. In most cases, *M. tuberculosis* can be eliminated or controlled, resulting in latent infection with no visible symptoms [55]. However, an infection can occur that progresses to the active form of tuberculosis in immunosuppressed individuals [56]. TB has been considered a global public health emergency since 1993 by the World Health Organization [57]. Currently, the situation has worsened due to the emergence of multi- and extensively drug-resistant human immunodeficiency virus (HIV)-TB co-infection and type 2 diabetes epidemic.

#### 5.1.1. Current Treatment and Drawbacks

The classical regimen of treatment against tuberculosis is based on “first line” antibiotics: rifampin, isoniazid, ethambutol, pyrazinamide, or streptomycin, which must be administered for at least 6 months. In addition to the long time of treatment and the secondary effects of some drugs, the bacterium has developed resistance or multidrug resistance. Treatment for people diagnosed with rifampicin-resistant TB (RR-TB), isoniazid-resistant TB, and multidrug-resistant TB (MDR-TB, defined as resistance to isoniazid and rifampicin) requires regimens that include second-line drugs, such as bedaquiline and fluoroquinolones; these regimens are more expensive (≥USD 1000 per person) and cause more side effects than first-line treatments for drug-susceptible tuberculosis. Pre-extensively drug-resistant TB (pre-XDR-TB, defined as TB that is resistant to rifampicin and any fluoroquinolone) and XDR-TB (resistance to rifampicin, any fluoroquinolone, and at least one of bedaquiline or linezolid) are even harder to treat [52].

The phenomenon of drug resistance remarks the urgent necessity of structurally new and potent anti-TB agents. A summary of all new anti-tuberculosis agents has been compilated, reporting 35 new structural classes including benzothiazoles, coumarins, dihydropyridines, isoxazoles, mycobactin-artimisinin conjugates, and thiourea derivatives, among others [58,59]. Most of drugs launched in the last decade were derived by modifying known drugs or lead structures.

#### 5.1.2. Methodological Approach for the Search of Anti-*M. tuberculosis* Compounds

All the criteria to take into account for new drug development against infectious diseases such as tuberculosis were summarized in 2015 [24]. These criteria were applied to all compounds described here, including those reported for the first time, and include the following: (1) The minimum inhibitory concentration (MIC), that is, the lowest drug concentration that inhibits more than 99% of *M. tuberculosis* growth on solid Middlebrook medium within 21 days of incubation at 37 °C. Growth was obtained by using REMA (resazurin microtiter assay) [60]. (2) The 50% cytotoxic concentration (CC_50_) on VERO cells, determined with a colorimetric assay using MMT (3-[4,5-dimethylthiazol-2-yl]-2,5 diphenyl tetrazolium bromide) to evaluate the cytotoxicity of the drug on mammalian cells [61]. (3) The selectivity index (SI), a CC_50_/MIC ratio that measures the window between cytotoxicity and antimicrobial activity. Values of the tested compounds were obtained against mono-resistant tuberculosis strain H37Rv.

Regarding our work in identifying new anti-tuberculosis drugs, the first strategy was mostly based on modifying known drugs or lead structures. In this regard, we reported in 2019 the synthesis of antitubercular N-[5-(4-chlorophenyl)-1,3,4-oxadiazol-2-yl]-(nitroheteroaryl) carboxamides, as well as the activity of acylthiosemicarbazides [62] and (2Z)-3-hydroxy-3-(4-R-phenyl)-prop-2-enedithioic acids [63]. Now, we are exploring the analysis of molecular docking or medicinal chemistry to choose lead molecules against a specific tuberculosis target.

#### 5.1.3. Compounds Tested against *M. tuberculosis*

To date, the consortium has evaluated 273 compounds obtained by chemical synthesis and 254 natural compounds against *M. tuberculosis* (some of them reported for the first time in this paper), of which 13 and 3, respectively, have been considered hits (Table 2) [24].

### 5.2. Chagas Disease (American Trypanosomiasis) as a Target of the NUATEI Program

American trypanosomiasis, which evolves into Chagas disease, is a public health problem that affects between 6 and 7 million people, mainly in Latin America [4]. This disease is caused by the hemoflagellate protozoan *Trypanosoma cruzi*, which is transmitted to different mammals, including humans. After the discovery of this disease, it was believed to be endemic to Brazil; however, over the decades, cases have been reported throughout South America, Central America, México, and the southern United States [65]. Humans can become infected in several ways: orally, congenitally, through the transfusion of contaminated blood or organ transplantation, and via laboratory accidents; however, the main pathway is vector transmission [66].

Chagas disease can manifest itself in two phases: acute and chronic. In the acute phase, parasitemia is detectable in blood samples under the microscope, and the symptoms are usually mild and non-specific, such as fever, malaise, hepatosplenomegaly, and atypical lymphocytosis. Sometimes, a skin nodule (chagoma) or prolonged painless eyelid swelling (Romaña sign) may be present at the inoculation site. Most acute infections are never detected [67]. In less than 1% of infections, the acute phase is severe and life-threatening due to meningoencephalitis or myocarditis. This more severe form includes fulminant myocarditis that results in a wide range of electrocardiographic and echocardiographic abnormalities and even death secondary to congestive heart failure [68]. The acute phase lasts approximately 6 to 8 weeks.

Symptoms of the acute phase resolve in the subsequent weeks to months after infection. Once this happens, the person enters a chronic asymptomatic or indeterminate phase of Chagas disease, characterized by undetectable parasitemia as the parasites invade target tissues. Specific antibodies against *T. cruzi* develop; however, this response does not eliminate the parasite, and for most patients, this indeterminate phase is lifelong. The chronic symptomatic phase appears years or even decades later when about 30% progress to detectable organ damage. The most serious manifestation is cardiomyopathy, which can be seen in up to 20–30% of all cases, followed by damage to the digestive system in 5–20% and a mixed form (cardiac and digestive) in 5–20% [65]. The characteristics of Chagasic cardiomyopathy are variable, the most common finding being dilated cardiomyopathy accompanied by abnormalities of the conduction system. Electrical manifestations include right bundle branch block, left anterior fascicular block, sinus node dysfunction, and ventricular arrhythmias. Structural abnormalities may include left ventricular aneurysms and secondary embolism because of thrombus formation and heart failure [69]. Diagnosis is confirmed with positive serology, detection of the parasite via xenodiagnosis, blood culture, or polymerase chain reaction (PCR).

#### 5.2.1. Current Treatment and Drawbacks

The drugs currently available for the treatment of Chagas disease are nifurtimox and benznidazole. These were introduced over 50 years ago. Benznidazole (Bz) is a nitroheterocyclic compound that contains a nitro group attached to an imidazole ring, which is activated by the parasite’s nitroreductase (NTR), generating a highly reactive compound that has cytotoxic activity against Trypanosomes [70]. On the other hand, nifurtimox (Nfx) is attributed to the ability to induce a maximum stimulation of O2^•−^ (superoxide anion) and to initiate the diffusion of hydrogen peroxide (H_2_O_2_) toward the parasites; therefore, the trypanocidal action of Nfx is mediated by the reduced products of oxygen [71]. The efficacy of Nfx and Bz is restricted to the disease’s initial phases and is unsuitable for treating chronic cases.

However, frequent adverse effects are observed in the use of Nfx, such as anorexia, loss of weight, psychic alterations, excitability, sleepiness, digestive manifestations such as nausea or vomiting, and, occasionally, intestinal colic and diarrhea. In the case of Bz, skin manifestations are the most notorious (e.g., hypersensitivity, dermatitis with cutaneous eruptions, generalized edema, fever, lymphadenopathy, articular and muscular pain), with depression of bone marrow, thrombocytopenic purpura and agranulocytosis being the more severe manifestations [45]. In addition, there is a correlation between the genetic characteristics of the parasite and resistance to Bz, which makes some parasite strains more resistant to the drug [72]. In the case of Bz, this resistance can be partially explained by the genome-wide accumulation of mutations in the resistant parasites, in addition to variations in DNA copy number. Mutations in DNA repair genes were observed to be linked with increased susceptibility to DNA alkylating and inter-strand crosslinking agents [73].

#### 5.2.2. Methodological Approach for the Search of Anti-*T. cruzi* Compounds

All methodologies for finding new molecules against *T. cruzi* are reviewed in [74], including the methodology for the compound reported here for the first time. In this consortium, pure active principles from plants obtained by chromatographic methods have been tested against *T. cruzi* using several approaches: reduction in the number of parasites by counting under optical microscopy, changes in metabolic activity through MTT assays, variations in parasite morphology using Giemsa staining and light microscopy, and ultrastructure analysis by transmission electron microscopy, as well as the determination of reactive oxygen species (ROS) using flow cytometry. Likewise, the cytotoxicity of the compounds has been determined through MTT assays using VERO cells to obtain the CC_50_ (mean cytotoxic concentration), a methodology that has been previously described [75,76]. The concentration of compound necessary to reduce the growth or metabolic activity of *T. cruzi* by 50 percent (IC_50_) is determined by testing a wide range of concentrations (3–400 µM) during 6 to 48 h incubation [75,76]. Another approach includes the analysis of the effect of new synthetic molecules based on previous FDA-approved drugs, as well as the effect of endophyte metabolites, by employing the same assays described above. Some of the compounds successfully tested in vitro have been used in in vivo assays with murine models infected with a virulent strain of *T. cruzi*. In combination with suboptimal doses of benznidazole (the drug of choice in the current treatment), the compounds reduced parasitemia and allowed the survival of mice (submitted for publication). 

#### 5.2.3. Compounds Tested against *T. cruzi*

Within the NUATEI consortium, our group has analyzed the effect of more than 60 different molecules, including natural and synthetic drugs, against the parasite’s epimastigote and trypomastigote phases (infective phase). Some of them are shown in Table 3. We also collaboratively conducted computational studies to identify several potential compounds with trypanocidal activity [77].

Of the compounds in this table, it can be mentioned that some, such as the case of the Mammea A/BA compound from *C. brasiliense*, have also been shown to induce morphostructural changes and oxidative stress in the epimastigotes of *T. cruzi* associated with apoptonecrosis [76]. On the other hand, of the in vivo assays, it is worth mentioning the one carried out to evaluate the therapeutic properties of the amphotericin B derivative A21. The test was performed by administering various doses of the compound, alone or in combination with benznidazole, for 22 consecutive days to mice intraperitoneally infected with a virulent strain of *T. cruzi*. The compound was administered twice a day (every 12 h) intraperitoneally from day 1 post-infection. The presence of parasites in the blood (parasitemia) was evaluated every third day, the weight of the animals registered every week, and the mortality of infected animals every day. Unlike benznidazole, A21 reduced the parasite load in the blood but did not prevent the death of mice. However, the combination of A21 with benznidazole reduced parasitemia and prevented the death of infected mice for up to 90 days, whereas mice infected and treated with the compounds independently did not survive more than 22 days post-infection. The use of A21 in combination with benznidazole to treat Chagas disease in humans is protected by a patent [81].

### 5.3. Amoebiasis by Entamoeba histolytica as a Target of the NUATEI Program

*Entamoeba histolytica* is the protozoan parasite that causes amoebiasis in humans. According to the WHO, there are around 500 million infected people in the world, and it is estimated that only 10% develop the disease. Intestinal disease is estimated to affect 50 million people annually, causing between 40,000 to 100,000 deaths, which places amoebiasis as the third cause of death from parasitic diseases in the world and the first from an enteric parasite [6]. In a global burden of diseases study from 1990 to 2010, worldwide deaths due to amoebiasis were reported to be around 1 per 100,000 people, causing a loss of disability life years (DALY) of 32 years on average [82]. According to the Mexican General Directorate of Epidemiology, amoebiasis is the most common parasitosis in this country, with at least 200,000 cases reported annually in the last decade [83].

The life cycle of *E. histolytica* consists of two morphologically well-differentiated stages: the trophozoite, or vegetative and invasive form, and the cyst or resistant and infective form. The infection begins with the ingestion of water or food contaminated with mature cysts from human feces. Thanks to their chitin cover, ingested cysts can resist the effect of gastric juices until they reach the lower part of the small intestine, where they are excysted, losing their cover. Next, a tetranucleated amoeba is released that duplicates its nuclei, followed by cytoplasmic divisions that result in the formation of eight small amoebas called metacystic amebulae [84]. Amebulae migrate to the large intestine, where they become mature trophozoites that adhere to and colonize the intestinal mucosa. There, the trophozoites feed on bacteria and cell debris, reproduce themselves by binary fission and, in some cases, penetrate the mucosa, causing lesions of different magnitudes and intestinal symptomatology. Sporadically, trophozoites migrate to other organs, mainly the liver, which becomes necrotic, and form abscesses [6].

#### 5.3.1. Current Treatment and Drawbacks

Amoebiasis is a disease associated with poverty, overcrowding, and poor hygiene habits, so only people who live in endemic areas or travel to them are at risk [85]. Once diagnosed, a well-established treatment plan should be followed. Throughout the history of the disease, numerous drugs have been used to treat it, including compounds that have even been withdrawn from the market due to their high toxicity. In general, anti-amoebics can act at the luminal level for treating amoebic non-dysenteric colitis (e.g., paromomycin, diiodohydroxyquinoline, and diloxanide), at the systemic level for treating invasive amoebiasis (e.g., nitroimidazoles), or both. The 5-nitroimidazoles, including metronidazole, tinidazole, ornidazole, and secnidazole, are the most used drugs for their high efficiency, being indicated for patients with symptomatic intestinal amoebiasis and extraintestinal amoebic liver abscesses. Of them, metronidazole is the drug of choice due to its potent amoebicidal effect and low cost [86]. However, all of them have severe drawbacks, such as nausea, vomiting, diarrhea, gastritis, headache, leukopenia, and allergy, which usually condition their abandonment prior to the eradication of the infection [87]. Metronidazole genotoxicity in human cells, mutagenicity in bacteria, and carcinogenicity in rodents have also been reported [86,88,89]. On the other hand, its indiscriminate use, as it is prescribed even in asymptomatic cases, seems to contribute to the development of metronidazole-resistant parasites [89,90,91]. In addition, metronidazole is unable to kill the cyst stage of *E. histolytica*, responsible for the transmission of the disease, making necessary a follow-up treatment with an additional drug. Therefore, better efficiency has been suggested for a therapy combining metronidazole with a luminal amoebicidal [87,92].

Regarding other compounds, some results suggest that tinidazole may be more effective and cause fewer adverse events than metronidazole. Nevertheless, metronidazole is still the drug of choice for amoebic dysentery [87]. Nitazoxanide, a nitrothiazolyl-salicylamide derivative, seems to be more effective than metronidazole in curing intestinal amoebiasis (70–90% cure rate) [93]. Unfortunately, the results are contradictory [3,87,94]. Instead, paramomycin is used to treat asymptomatic carriers [89], and diloxanide furoate is only 51% effective [87,95,96]. Quinfamide and compounds of the diiodohydroxyquinoline group act in the intestinal lumen and are absorbed at minimal levels, making them not effective against invasive amoebiasis [97]. Furthermore, diiodohydroxyquinolins cause serious and irreversible optic atrophy, blindness, and neuropathy [98,99].

In the last decade, a high-throughput screen was performed to identify anti-amoebic drugs. The approach allowed the identification of auranofin, a rheumatoid arthritis drug, as highly active [100], targeting the parasite ROS detoxifying system [101].

#### 5.3.2. Methodological Approach for the Search of Anti-Amoebic Compounds

Details about the methodologies that we have used to evaluate the effect of compounds on *E. histolytica*, including those reported for the first time here, can be found in works previously reported by our group [102,103,104,105,106,107]. In brief, the anti-amoebic effect of the compounds was evaluated in vitro on trophozoite cultures of the *E. histolytica* HM1:IMSS strain. This strain is the most used worldwide for studies related to the biology of this parasite. Extracts and compounds prepared in solvents are added to trophozoite cultures in a concentration range from 0.01 μg/mL to 100 μg/mL, with an initial amount of 1–2 × 10^4^ trophozoites/mL, in tubes or 96-well plates (200 μL/well). After 24 h of incubation, the amoebae viability is determined using MTT. Untreated and vehicle controls (solvent where the sample was prepared) are included, and the IC_50_ is calculated from the data obtained. In the most outstanding cases, the mechanism of death induced by the amoeba (apoptosis and/or necrosis) is determined.

In the case of compounds with an IC_50_ very close to, or similar to, metronidazole, their therapeutic potential to treat amoebiasis is evaluated in in vivo experimental models of intestinal and extraintestinal amoebiasis. To determine the therapeutic effect of the compounds on intestinal amoebiasis, mice of the C3H/HeJ strain are infected with the amoebas at the level of the cecum by means of a laparotomy. Once the infection is established (two weeks post-exposure), the animals are treated orally with different doses of the candidate compounds daily for 4–7 days using a gastric tube. A group of control mice is treated orally with the vehicle in which the compound to be evaluated has been prepared (sham group). After the completion of the 21-day experimental period (infection plus treatment), all mice are sacrificed on day 22 using excess anesthesia. Post-mortem, the ceca of the animals are excised and processed for histology studies on H&E-stained tissue sections. The degree of infection is determined by the presence and number of amoebae per tissue area and by the observation of parasites in the ceca of the animals. The level of infection remission is statistically analyzed using Fischer’s exact test [102].

For the evaluation of the therapeutic effect of compounds on extraintestinal amoebiasis, a model of liver abscesses in golden hamsters (*Mesocricetus auratus*) is used. Briefly, after anesthesia, the hamsters are subjected to an aseptic laparotomy whereby the portal vein is exposed, into which the *E. histolytica* trophozoites are injected (10^6^ virulent trophozoites). Newly infected hamsters are treated intraperitoneally with different doses of the extracts or compounds generally for 1 week, after which the animals are sacrificed and the livers excised. The extent of amoebic abscesses is recorded as hepatomegaly (weight of the livers) and evaluated in histological sections stained with H&E [103].

#### 5.3.3. Compounds Tested against *E. histolytica*

Overall, we have evaluated the anti-amoebic in vitro activity of more than 170 compounds and plant extracts. Of these, the IC_50_ of 77 samples has been determined, of which 11 can be considered highly active because they exhibited an IC_50_ close to that of metronidazole (≤10 μg/mL), 25 can be considered moderately active (IC_50_ between 10 and 100 μg/mL), and the rest can be considered inactive (IC_50_ > 100 μg/mL). Below is a table with the IC_50_ of the hit compounds (Table 4).

Of the 11 highly active compounds, three were evaluated in the hamster model of amoebic liver abscesses and four in the murine model of intestinal amoebiasis. Intraperitoneal treatment with extracts from the plant *Tabernaemontana arborea* and its alkaloids ibogaine and voacangine did not affect the development of hamsters’ amoebic liver abscesses, even though the compounds readily killed amoeba in vitro through apoptonecrosis [104]. In contrast, oral treatment for 5 days with 20 mg/kg of bovine lactoferrin eradicated intestinal infection in 63% of infected mice while reducing the number of trophozoites in the remaining animals [106]. In this case, in addition to killing the parasite through necrosis, the elimination of amoebae in the intestine also correlated with changes in the pro-inflammatory environment, suggesting an immunomodulatory effect of lactoferrin. On the other hand, an oral treatment for 5 days with 10 mg/kg of peptides derived from lactoferrin, lactoferrampin, lactoferricin B, and lactoferricin 17–30 resolved the infection in 100, 75, and 75% of mice, respectively, killing amoebas by necrosis [107].

### 5.4. HIV-1 as a Target of the NUATEI Program

Human immunodeficiency virus type 1 (HIV-1) is an enveloped virus that has a special tropism for cells of the immune system. The virus causes an acute infection that subsequently persists as a largely asymptomatic disease in most infected individuals, however causing deleterious cumulative effects on the immune system, which manifest as acquired immunodeficiency syndrome (AIDS) after a variable period ranging from a few to 10 or more years. By the end of 2022, around 39 million people were living with HIV-1 worldwide [108]. In Mexico, 361,095 people have been diagnosed with HIV-1, of which 112,578 (31.2%) have died. An estimated 20,000 new cases continue to occur each year in this country [8].

HIV-1 preferentially infects cells of the immune system, particularly CD4+ T lymphocytes and monocytes. The early infection of the gut-associated immune tissue causes inflammation and functional alteration of the intestinal lumen epithelium, which otherwise acts as a barrier against bacterial antigens. The damage then allows the passage of bacterial molecules to underlying tissues, providing antigenic stimulation that results in permanent immunological activation, further promoting virus replication in activated lymphocytic cells. Subsequent damage to other lymphoid tissues causes the gradual loss of the efficiency of the adaptive immune response. The long-term maintenance of the virus in cellular reservoirs makes it difficult to eradicate the infection. The virus-specific receptors are the CD4 molecule and the CCR5 and CXCR4 chemokine receptors. The detection of viruses that interact with CXCR4 in the blood of patients is associated with the advent of AIDS [109]. Currently available drugs against HIV-1 control it but do not eliminate the infectious agent, are expensive, and still cause significant clinical side effects, making new treatments a constant need [110].

#### 5.4.1. Methodological Approach for the Search of HIV Inhibitors

In the search for specific inhibitors of the virus, we test the effect of candidate compounds on the activity of HIV reverse transcriptase and virus replication in cell cultures. Inhibition of reverse transcription is determined in a DNA synthesis assay from oligonucleotides (Oligo dT and Poly A) using the EnzChek Reverse Transcriptase Assay Kit (Thermo Fisher Scientific, Waltham, MA, USA). In this assay, the PicoGreen reagent detects RNA-DNA complexes formed from the oligonucleotides. The assay is performed in 96-well plates using nanomolar to micromolar concentrations of the compounds of interest. The assay generates a fluorescent signal that is read under standard conditions for fluorescein in a microplate reader.

The effect of compounds on HIV-1 replication uses the viral strains IIIB and MN of HIV-1, as well as primary isolates propagated in the lymphoblastic cell lines H9 and MT-2. Determination of infectious units in infected cell supernatants and the inhibition of HIV-1 replication are performed through the infection of the JLTRG reporter cells [111]. JLTRG cells are human CD4+ T lymphocytes derived from the Jurkat cell line and contain the green fluorescent protein (GFP) gene under the control of the virus promoter known as long terminal repeats (LTR). When these cells are infected with HIV-1, the viral protein Tat binds to the LTRs and activates GFP synthesis. Green fluorescence in infected cells can be quantified using flow cytometry. The cells and virus are donated by the AIDS Reagent Program of the United States National Institutes of Health.

#### 5.4.2. New Synthetic Pyridinones against HIV-1 Reverse Transcriptase

Inhibitors of the HIV reverse transcriptase (RT) are classified into two classes: nucleoside-type reverse transcriptase inhibitors (NRTIs), which bind directly to the active site, and non-nucleoside reverse transcriptase inhibitors (NNRTIs), which bind to a hydrophobic site located 10 Å from the active site, called the allosteric site. A family of pyridinone-type compounds has been synthesized in our group on the base of their binding in silico to the allosteric site of the reverse transcriptase. Different studies have shown that the pyridinone core can serve as a basis for the search for new compounds active against HIV-1 strains sensitive and resistant to current drugs. The flexibility of the binding of pyridinone-derived NNRTIs to the allosteric site of the HIV-1 RT provides them the ability to acquire different conformations, which may be related to their enhanced effect against virus mutations. This results in improved activity even against HIV-1 strains resistant to first-generation drugs such as Nevirapine and Efavirenz. In particular, two groups reported the crystal structure of RT in complex with three pyridinone derivatives [112,113]. They described how substitutions of the pyridinone skeleton affect the binding and activity of these inhibitors to variants of the enzyme-containing resistance mutations. In silico design studies of INNSTRs with numerous variants of the pyridinone chemical core have been performed at the UNAM Faculty of Chemistry, particularly employing quantitative structure–activity analysis (QSAR) modeling using the k-nearest neighbor system, which uses multiple descriptors derived from the two-dimensional molecular topology of RT–pyridinone complexes. The modeling process has been extensively validated and applied to the prediction of the activity of compounds that were not used in the model development against the RT. Together with flexible molecular docking structural studies, QSAR models have been used to describe the features of the interaction of pyridinone-type compounds with the allosteric site of RT [114,115,116,117,118].

To date, there are 32 newly synthesized pyridinone-derived compounds with the potential to inhibit the HIV-1 reverse transcriptase enzyme. The chemical structure of the compounds was confirmed by nuclear magnetic resonance. The structure of some of these compounds and a description of their in silico interaction with the reverse transcriptase enzyme have already been reported [119]. Currently, our aim is to understand the structural basis of the interaction of pyridinone-type compounds with the allosteric site of the enzyme, as well as the chemical determinants of their antiviral activity in vitro. One compound (DG-9) inhibits the replication of the HIV-1 strain IIIB with a good IS and inhibits reverse transcriptase activity. DG-9 has shown minimal cytotoxic activity in JLTRG cells [120]. Likewise, specific substituents in the pyridinone ring associated with the cytotoxic effects have been identified. These observations support the hypothesis that computational structure–activity relationship models allow the design of molecules with the ability to inhibit reverse transcriptase activity and that hydrophobic substituents at specific positions of the pyridinone core are relevant to their biological activity. This hypothesis will be validated by the design of new compounds that combine the observed favorable features, followed by structural and biological activity evaluation. Currently, a new panel from compounds derived from the DG-9 structure is under study.

## 6. Concluding Remarks and Future Considerations

The discovery of new drugs for treating human infectious diseases is not only a scientific endeavor; it is a matter of public health, global security, and social justice. It remains a pressing and critical challenge for several compelling reasons, including drug resistance, treatment gaps, vaccine limitations, emerging and reemerging diseases, globalization, and biosecurity concerns, among others. This includes new drugs and repurposing existing drugs for new applications. Continued investment in research, development, and multidisciplinary collaboration is essential to meet these ongoing challenges and to be prepared for the infectious disease threats of the future. Despite all the advances that have been achieved so far for the control of infectious diseases, they continue to be a serious public health problem, mainly in developing countries. The impact of the prevalence of infectious diseases is also reflected in the economy of the countries, as they are responsible for work disability and excessive expenses in treatment and rehabilitation that affect the nation’s budget. In Mexico, according to the records of the Ministry of Health, conditions such as intestinal amoebiasis are among the 20 main causes of communicable diseases in the population, while cases of tuberculosis and Chagas disease are increasing, and those of AIDS persist [83]. In part, the problem lies in the development by infectious agents of various strategies and molecular mechanisms of resistance to drugs, as well as the abandonment or lack of adherence to treatments due to the powerful side effects they trigger in patients, and, finally, to the appearance of more virulent and/or innately less susceptible variants.

The NUATEI consortium, as presented in this manuscript, was established to bring together multidisciplinary research groups from UNAM and other institutions. The goal is to identify new compounds of natural or synthetic origin, primarily from plants, with the potential to treat common infections such as tuberculosis, amoebiasis, trypanosomiasis, and AIDS. This initiative aims to address the limited treatment options, high disease prevalence, and the emergence of drug resistance in the causative agents. Additional factors driving the need for new drugs include the lengthy treatment regimens, co-infections, and comorbidities for tuberculosis [121], variability in disease severity for amoebiasis [122], multiple genotypes and chronic phase neglect for trypanosomiasis [123], and co-infections and comorbidities for AIDS [7]. The NUATEI consortium’s efforts focus on finding new compounds with similar or superior efficacy to the current drugs while improving safety. To achieve this, specific targets are being identified through cellular and molecular approaches to design novel drugs. In addition to traditional search strategies, the consortium also stands in a new stage of antibiotic research supported by advancements in “omics” technologies. These strategies include genome mining, single-cell genome amplification, metabolomics, and proteomining [124,125].

So far, the NUATEI consortium has made remarkable progress in discovering more than a dozen compounds, primarily derived from plants, with the potential to treat diseases caused by the microbial agents under study. One of these compounds has been recognized as patent-worthy for its effectiveness against trypanosomiasis [81]. In preclinical trials using murine models (Chagas and amoebiasis), combinations of some hit compounds with the choice treatments (e.g., A21 with benznidazole or metronidazole) will be used within liposomes for better delivery. Virus-like particles as carriers will also be studied in the future [126].

However, there is still much to be accomplished, including testing most of these compounds in animal models and conducting thorough safety evaluations in mammals, if not humans. Likewise, we are looking for the identification of more compounds with antimicrobial activity through high-throughput screening on large compound libraries using in silico analysis. Ultimately, we aspire for this collaborative effort to mark a substantial milestone in research, leading to the development of a new generation of compounds with microbicidal properties to combat four of the most prevalent infectious diseases in the Mexican population.

## Figures and Tables

**Table 1:** Infectious agents targeted by the NUATEI Program and their local and global impact on human health.

Infectious Agent	Type	Disease	No. Cases (Mex./Worldwide)	Annual Deaths	Mortality Ranking	Refs.
*Mycobacterium tuberculosis*	Mainly pulmonary bacteria	Tuberculosis	25,449 in 2016/ 10.4 million	1.6 million	9th worldwide	[3]
*Trypanosma cruzi*	Blood and visceral protozoan	Chagas disease	1.1 million in 2016/ 6–7 million	10,000 to 12,000	4th among protozoan parasites	[4,5]
*Entamoeba histolytica*	Enteric and hepatic protozoan	Amoebiasis	200,000 in 2019/ 50 million	40,000 to 100,000	3rd among protozoan parasites	[6]
Human immunodeficiency virus (HIV)	Systemic virus	AIDS	13,489 in 2023/ 39 million	480,000 to 880,000	19th worldwide	[7,8]

**Table 2:** Compounds with activity against *Mycobacterium tuberculosis* in the framework of the consortium NUATEI.

Compound	Source	*M. tuberculosis* Strain	MIC_100_ (mg/mL)	CC_50_ (mg/mL)	SI	Reference
GPC-1	Synthetic	H37Rv	7.8	106	13.6	[63]
ARO-1	Synthetic	H37Rv	7.8	129	16.5	[64]
SB-38	Synthetic	H37Rv	3.9	298	76.4	This paper
AMBH-4	Synthetic	H37Rv	31.25	387	12.3	This paper
R1-4F-2	Synthetic	H37Rv	15.6	219	14.0	This paper
SB-18	Synthetic	H37Rv	7.8	138	17.7	This paper
Benzo-NO2	Synthetic	H37Rv	31.25	555	17.76	This paper
R1-4Br-2	Synthetic	H37Rv	15.6	288	18.5	This paper
F-NO2	Synthetic	H37Rv	31.25	696	22.2	This paper
R1-4I-2	Synthetic	H37Rv	15.6	451	28.9	This paper
RCS-NO2	Synthetic	H37Rv	7.8	255	32.7	This paper
Anthracycline StefB	Endophyte from *Amphipterygium adstringens.*	H37Rv	7.8	99.3	12.7	[59]
Anthracycline StefB	Endophyte from *Amphipterygium adstringens.*	Strain 209 * (clinic isolated)	3.9	99.3	25.46	[59]

Hits against *M. tuberculosis* H37Rv ATCC 27294 (SI > 10). * Rifampicine resistant strain. SI = selectivity index was obtained using SI = CC_50_/MIC_100_. CC_50_: Obtained from testing the effect of the compounds on VERO cells at 24 h [24]. This paper = data showed for the first time in this publication.

**Table 3:** Compounds of natural and synthetic origin that have been tested against *T. cruzi* in the framework of the NUATEI consortium.

Natural Origin	Compound Name	IC_50_ (μM)	SI	Reference
*Cnidoscolus spinosus*	GDL-1	>200	ND	[78]
*Mikania* sp.	GDL-10	<50	ND	[78]
	GDL-15	<50		
*Ambrosia* sp.	GDL-21	<50	ND	[78]
*Calophyllum brasiliense*	Mix of coumarins	22.5	ND	[76]
*Calophyllum brasiliense*	Mammea A/BA	17.6	7.7	[76]
*Parthenium hysterophorus*	Ambrosin	68.4	11.46	[79]
*Decachaeta incompta*	Incomptine B	132.3	8.42	[79]
*Vernonia liatroides*	Glaucolide E	199.7	2.37	[79]
*Streptomyces* spp.	Thiostrepton	4.5	8.8	[80]
Synthetic Compounds				
Amphotericin B	A21	<2	>200	[81]
5-nitroimidazoles	Secnidazol	>300	ND	This paper
	Co-secnidazol	>300		
Variable chemical nature	1, 2, 3, 5, 6, 7, 12, V-5, M1A, L1A, J1B, 93, F42, A4, lan, Ffan, Fan	All >50	ND	This paper
Biometal compound	PtSO_3_	>100	ND	This paper
Biometal compound	Risedronato-Zn	<25	ND	This paper
Biometal compound	Risedronato-CuIA	<2	1.09	This paper
Biometal compound	Risedronato-CuIB	<2	1.23	This paper

This paper = data showed for the first time in this publication. ND: not done. SI = selectivity index was obtained using SI = CC_50_/IC_50_. CC_50_: Obtained from testing the effect of the compounds on VERO cells at 24 h [24].

**Table 4:** Natural compounds identified by the consortium NUATEI as hits against *E. histolytica* trophozoites.

Source	Compound or Fraction	IC_50_ at 24 h (μg/mL)	SI	References
*Tabernaemontana arborea* (plant)	Alkaloid fraction	0.2	10.2	[104]
	Ibogaine	0.8	252.8	
	Voacangine	0.8	13.6	
	Voacamine	10	ND	
*Annona purpurea* (plant)	Alkaloid fraction	20.8	ND	[105]
	Glaziovine	9.9	ND	
	3-Hydroxiglaucine	66.6	ND	
	Norpurpureine	68.5	ND	
Cow milk	Lactoferrin (Lf)	2	>1000	[106]
Synthetic peptides	Lactoferrampin	0.5	ND	[107]
	Lactoferricin 17–30	1	>1000	
	Lactoferricin B	1	>1000	
Synthetic compound	A21 (amphotericin B derivative)	<1	>200	This paper
Synthetic compound	PRO54	0.8	109.25	This paper
Synthetic nitroimidazole	Metronidazol	1.2	>854.7	[107]

SI = selectivity index was obtained using SI = CC_50_/IC_50_. CC_50_: Obtained from testing the effect of the compounds on VERO cells at 24 h [24].

## Data Availability

The original contributions presented in the study are included in the article, further inquiries can be directed to the corresponding author.
